# On the Failure of Crankshafts in Thermoelectric Power Plants: Multiaxial Fatigue Analysis and a Comparative Survey on Crack Growth Threshold $\Delta K_{th}$

**DOI:** 10.3390/ma18174034

**Published:** 2025-08-28

**Authors:** Tiago Lima Castro, Thiago Abreu Peixoto, João Araujo Alves, Marcos Venicius Pereira

**Affiliations:** 1 Department of Metallurgical and Materials Engineering, Federal University of Rio de Janeiro—UFRJ, Avenida Horácio Macedo, 2030, Rio de Janeiro 21941-598, RJ, Brazil; tiago.castro@metalmat.ufrj.br; 2 Department of Mechanical Engineering, ENEVA S.A—Power Generation, Praia de Botafogo, 501, Torre Corcovado, Rio de Janeiro 22250-040, RJ, Brazil; thiago.peixoto@eneva.com.br; 3 Department of Chemical and Materials Engineering, Pontifical Catholic University of Rio de Janeiro—PUC-Rio, Rua Marquês de São Vicente, 225, Rio de Janeiro 22451-900, RJ, Brazil; joaoaraujo667@gmail.com

**Keywords:** critical plane-based models, stress intensity factor threshold range, failures in thermoelectric power plants, DIN 34CrNiMo6, DIN 42CrMo4, SAE 4140 and SAE 4340 steels, infinite fatigue life, Findley, Matake, McDiarmid and modified Wöhler curve method (MWCM)

## Abstract

Despite being designed considering infinite fatigue-life, failures of motor crankshafts forged from DIN 34CrNiMo6 steels have been reported in Brazilian power plants. As such, the present work aims to discuss the failure of a crankshaft within this context, with the purpose of verifying whether the stresses developed in critical locations of the component were in accordance with the steel’s fatigue limits, as well as if the material exhibits an adequate resistance to crack propagation. Taking into consideration a set of critical-plane stress-based multiaxial fatigue criteria, namely Findley, Matake, McDiarmid and Susmel and Lazzarin, the fatigue behaviour of the material is analysed and discussed. Furthermore, da/dN versus ΔK experiments were carried out with the purpose of determining the DIN 34CrNiMo6 steel’s crack growth threshold $\Delta K_{th}$ and comparing it to the $\Delta K_{th}$ of three other commercially available steels (DIN 42CrMo4, SAE 4140 and SAE 4340). The selected multiaxial fatigue criteria indicated that the stresses developed throughout the component were not sufficient to drive the crankshaft to failure, thus indicating safety. On the other hand, the DIN 34CrNiMo6 steel presented the lowest $\Delta K_{th}$ ($6.6\;MPa\;m^{1/2}$) among all the considered steels (10.86, 12.38 and $7.22\;MPa\;m^{1/2}$ for the DIN 42CrMo4, SAE 4140 and SAE 4340, respectively), therefore being susceptible to shorter fatigue lives in comparison to the other materials.

## 1. Introduction

In the past few years, thermoelectric power generation has been firmly positioning itself in the Brazilian energy matrix, becoming increasingly responsible for regular and substantial power dispatches. With the growing demand in the electric sector, the crankshafts of thermoelectric power plants (considered to be the main components of combustion engines) are being progressively subjected to longer service lives ($10^{8}$–$10^{12}$ loading cycles), therefore becoming susceptible to the risk of fatigue failures. Such a risk becomes more critical when taking into consideration the multiaxial nature of the loads (combination of bending, torsional and axial forces) to which the crankshafts are subjected to while in operation.

Between 2012 and 2017, at least twelve crankshaft failures (of gas or diesel engines) due to fatigue were reported in thermoelectric power plants [1]. Despite being designed to have infinite fatigue lives, i.e., decommissioning before failure, some of these failures occurred in crankshafts with one year of service or less [1]; these failures were attributed to microstructural heterogeneities and possible equipment design flaws.

In addition to requiring the replacement of the crankshaft itself, failures may also damage other engine components such as cylinders, connecting rods, bearings, etc., further increasing material losses, as well as resulting in indirect costs due to operational downtime, profit losses, contractual penalties and energy purchasing on the spot market. Therefore, an important technological barrier to be overcome by thermoelectric plants is the prevention of crankshaft failure during operation, thereby mitigating breakdown maintenance, material losses and unexpected expenses.

While in operation, the stresses developed in critical sites along the crankshaft constitute a set of non-trivial states of stress where, for each critical location, the components of the stress tensor vary independently in time throughout a loading cycle. As a consequence, fatigue behaviour analysis must take into consideration the complexity of the loading history, thus requiring the use of multiaxial fatigue theory [2].

Considering the nature of the operation, where stresses are expected to keep the crankshaft within the elastic regime, the stress-based approach has been commonly adopted for high-cycle fatigue analysis. The models pertaining to this group may be typically grouped as equivalent stress, stress invariants, average stress and critical plane stress [3], among others. For the case where non-trivial time-varying stress states are applied, critical plane models are effective as they manage to take into account complicated loading histories to determine which plane experiences the maximum damage due to fatigue, as well as its corresponding stresses. Fatigue behaviour is thus assessed by comparing the combined effect of the stresses acting on critical planes with the fatigue resistance limits of the material. As such, a set of critical plane-based models—namely Findley [4], Matake [5], McDiarmid [6] and Susmel and Lazzarin [7]—was selected with the purpose of addressing the fatigue behaviour of the crankshaft while in service. Accordingly, while several reviews on such models can be found in the literature [8,9], a comprehensive yet objective review of the selected criteria is provided in the next section of this paper.

**Figure 1:**
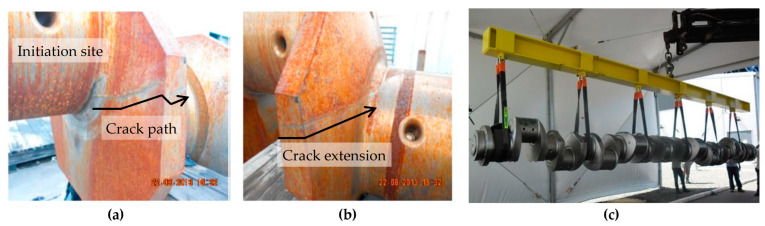
(**a**) Crack initiation site [10]; (**b**) crack extension [10]; (**c**) crankshaft replacement procedure in Brazilian thermoelectric power plants.

The research presents a technological contribution to thermoelectric power plants that use crankshafts/combustion engines in energy production. Therefore, the work demonstrated that the failure in question was not associated with design errors but with microstructural characteristics of the material, demonstrating the influence of the impurity content on the fatigue resistance of the components. As presented in Figure 1a,b, all failed crankshafts presented the same cracking pattern, where cracks were initiated at similar locations (henceforth addressed to as critical points), always in the presence of geometric features that promote the concentration of stress. Figure 1c depicts the hoisting of a crankshaft during breakdown maintenance.

## 2. Materials and Methods

**Figure 2:**
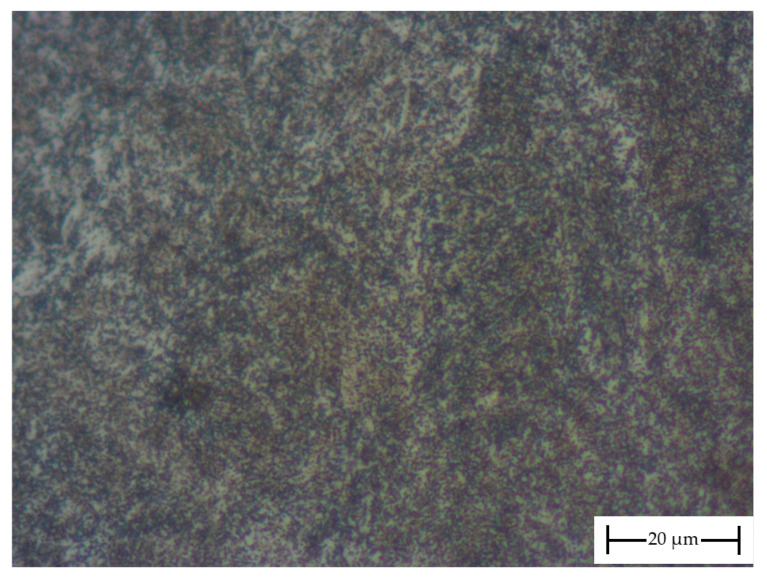
Micrograph of DIN 34CrNiMo6 steel, exhibiting a typical microstructure of tempered martensite and bainite.

The crankshaft considered in this work, which experienced premature failure due to fatigue while in operation, was forged from DIN 34CrNiMo6 steel. The failed component was removed from operation, sectioned into parts and a certain volume of the material was received. The component was quenched and tempered, exhibiting a typical microstructure of tempered martensite and bainite, as presented in Figure 2. The chemical composition and mechanical properties are presented in Table 1 and Table 2, respectively. Since one of the goals of the present work was to compare the crack growth resistance of DIN 34CrNiMo6 steel with other commercially available steels, Table 1 and Table 2 also include the data pertaining to the other materials involved in this work, namely DIN 42CrMo4, SAE 4140 and SAE 4340.

**Figure 3:**
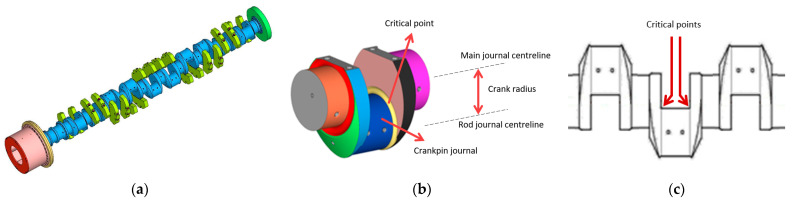
(**a**) Illustration of the crankshaft, presenting ten crankpin journals along its length [10]; (**b**) detailed view of a crankpin [10]; (**c**) location of the critical points on the crankpin journals.

The failed crankshaft, as illustrated in Figure 3a, comprised a total of ten crankpin journals. Each crankpin journal presented a couple of connecting rods that were connected to their corresponding pistons. As shown in Figure 3b, the crankpin journals are offset from the main journal’s centreline, thus describing circles of their own when the crankshaft is subjected to rotary motion.

The crankpin journals, which are numbered from 1 to 10, present a couple of critical points, as exhibited in Figure 3c, which are identified throughout the text as A01, B01, A02, B02 and so on, amounting to a total of twenty critical points.

**Figure 4:**
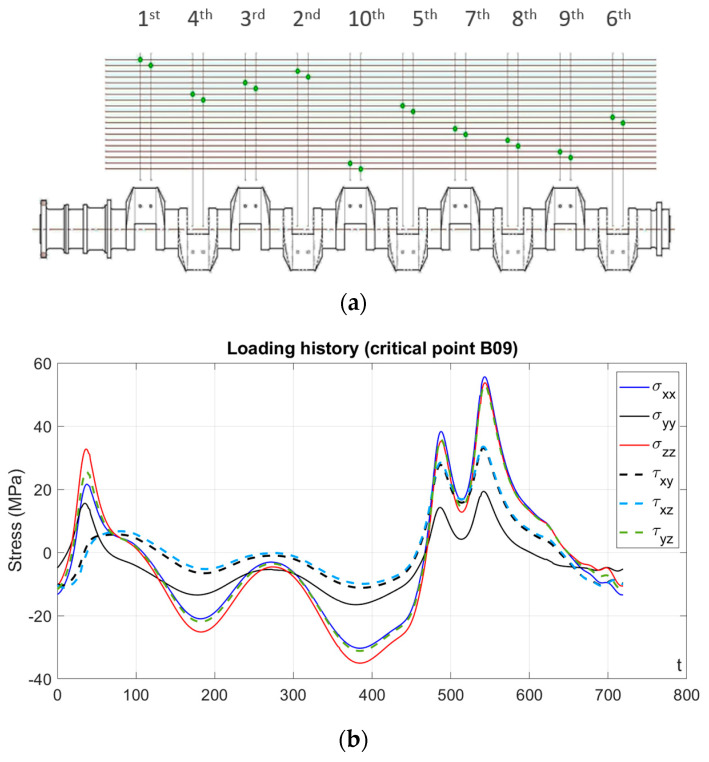
(**a**) Firing sequence; (**b**) estimated time-varying stress field relative to critical point B09, where each time unit corresponding to approximately $2.315\times 10^{-4}\;s$.

The crankshaft is put into motion due to a sequence of torques imposed to the component by the connecting rods due to the combustion reaction inside the cylinder bores. The sequence of firing, designed to ensure uniform rotation of the crankshaft, is presented in Figure 4a.

At this point, it is important to clarify that the research took into consideration (as input data) the results obtained from a finite element method (FEM) analysis carried out by an independent service provider. However, the methodology prior to the FEM analysis can be summarised as follows [11].

A cylinder from a similar engine, pertaining to the same thermoelectric plant, was instrumented with the purpose of measuring the pressure curves inside the cylinder bores under nominal power generation conditions (720 rpm). Such measurements were used to calibrate the physical–mathematical models relative to the engine’s thermodynamic processes using commercial software. Once the thermodynamic models were calibrated, the pressure curves for all twenty cylinders, i.e., the complete engine, were obtained via simulation. As such, based on the resulting pressure curves, as well as on the physical and geometric features of the crankshaft’s main components (pistons, rings, pins and connecting rods), the forces acting on the connecting rods and main bearings were estimated assuming an infinitely rigid shaft and with loads being applied by the connecting rod bearings. Finally, these forces were provided to the FEM team, who carried out the analysis to determine the time-varying states of stress developed along the crankshaft.

An example of the provided data is shown (as received) in Figure 4b and Table 3. The estimated stresses were seen to be repeated cyclically every two complete revolutions of the crankshaft, indicating a full loading cycle corresponded to 720°. Given that each increment in angular position corresponded to a given increment in time, stress data can therefore be presented either as function of the angular position or in terms of time units. Each time unit corresponds to approximately $2.315\times 10^{-4}\;s$, determined by considering 6 cycles of 720° per second (4320 °/s) and, accordingly, taking the inverse of this value.

As such, the fatigue behaviour of the crankshaft can be assessed with the use of critical-plane multiaxial high-cycle fatigue criteria. A set of models pertaining to this group, namely Findley (F), Matake (M), McDiarmid (McD) and Susmel and Lazzarin (S&L), was selected and applied to the estimated loading histories. The theoretical approach to these models considering the case of non-trivial stress states is fully described in Section 2.1 of this work.

In addition, the resistance against crack propagation $\Delta K_{th}$ of the DIN 34CrNiMo6 steel was experimentally measured and compared to those of the DIN 42CrMo4, SAE 4140 and SAE 4340 steels. Analogously, the methodology in question is fully described in Section 2.2.

### 2.1. Multiaxial Fatigue: Critical-Plane Stress-Based Models

In general terms, according to critical-plane stress-based multiaxial fatigue models, cracking is expected to initiate on the material planes where the maximum damage due to fatigue is experienced. Although the definition of a critical plane may vary depending on the selected criterion, critical plane identification first depends on evaluating the shear stress amplitude $\tau_{a}$ and the maximum value attained by the normal stress $\sigma_{n,max}$ within a loading cycle for a number of material planes intercepting the specimen. Once these values are known, the critical planes according to each model’s definition can be determined.

**Figure 5:**
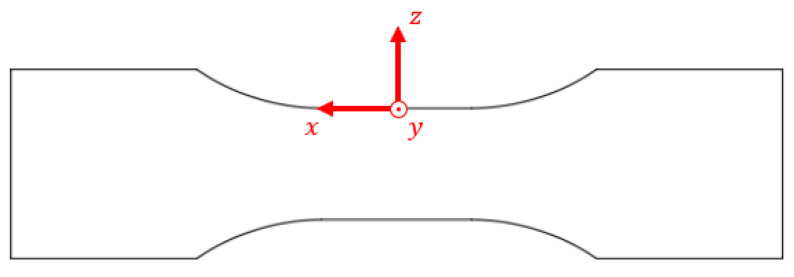
Specimen with the reference system positioned on its free surface, where cross-sectional area is at its minimum.

Taking into consideration the specimen shown in Figure 5, the reference system is positioned on its free surface, where the cross-sectional area is at its minimum [12], with the x-axis aligned with the longitudinal direction of the specimen and the z-axis aligned with the radial direction of the specimen.

**Figure 6:**
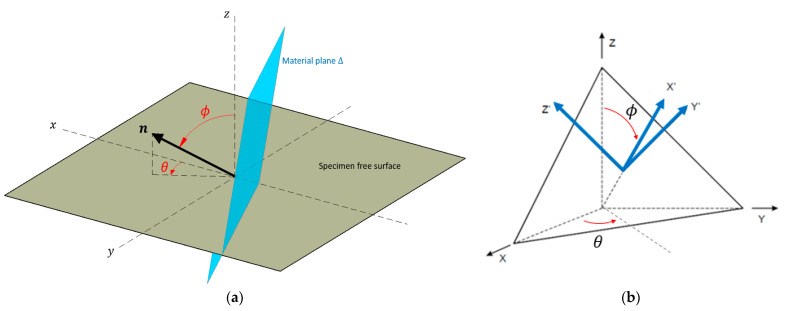
(**a**) Orientation of the material plane Δ in terms of the angles ϕ and θ; (**b**) reference system orientation after changing of basis.

A generic material plane Δ intercepting the specimen at its critical point is represented in Figure 6a. The orientation of the material plane Δ is uniquely determined by its normal unit vector n, described in spherical coordinates in terms of the azimuthal and polar angles $\left(\phi ,\theta \right)$, which are identified in Figure 6a [2].

The applied stress state is thus given by the symmetric stress tensor

(1) $$\sigma \left(t\right)=\left(\begin{array}{ccc} \sigma_{xx}\left(t\right) & \tau_{xy}\left(t\right) & \tau_{xz}\left(t\right)\\ \tau_{xy}\left(t\right) & \sigma_{yy}\left(t\right) & \tau_{yz}\left(t\right)\\ \tau_{xz}\left(t\right) & \tau_{yz}\left(t\right) & \sigma_{zz}\left(t\right) \end{array}\right),$$

where each of the 6 the independent components vary with respect to time. For generic orientations of the material plane Δ, changing the basis is convenient to describe the stresses acting upon it. Figure 6b presents the final orientation of the axes x’, y’ and z’ after changing the basis, where x’ corresponds to the normal direction, i.e., perpendicular to the material plane in question. The normal stress component and both the shear stress components acting on Δ are, respectively, given in [2]

(2) $$\sigma_{n}\left(t\right)=\sigma_{xx}a_{11}^{2}+\sigma_{yy}a_{12}^{2}+\sigma_{zz}a_{13}^{2}+2\left(\tau_{xy}a_{11}a_{12}+\tau_{xz}a_{11}a_{13}+\tau_{yz}a_{13}a_{12}\right)$$

(3) $$\tau_{x^{’}y^{’}}\left(t\right)=\sigma_{xx}a_{11}a_{21}+\sigma_{yy}a_{12}a_{22}+\sigma_{zz}a_{13}a_{23}+\tau_{xy}\left(a_{11}a_{22}+a_{12}a_{21}\right)\;+\tau_{yz}\left(a_{12}a_{23}+a_{13}a_{22}\right)+\tau_{xz}\left(a_{13}a_{21}+a_{11}a_{23}\right)$$

(4) $$\tau_{x^{’}z^{’}}\left(t\right)=\sigma_{xx}a_{11}a_{31}+\sigma_{yy}a_{12}a_{32}+\sigma_{zz}a_{13}a_{33}+\tau_{xy}\left(a_{11}a_{32}+a_{12}a_{31}\right)\;+\tau_{yz}\left(a_{12}a_{33}+a_{13}a_{32}\right)+\tau_{xz}\left(a_{13}a_{31}+a_{11}a_{33}\right),$$

where the above-mentioned auxiliary parameters ($a_{ij}$, with i,j=1,2,3) correspond to the components of the change of basis tensor Q, as given by [2]

(5) $$Q=\left(\begin{array}{ccc} a_{11} & a_{12} & a_{13}\\ a_{21} & a_{22} & a_{23}\\ a_{31} & a_{32} & a_{33} \end{array}\right)=\left(\begin{array}{ccc} \cos\left(\theta \right)\sin\left(\phi \right) & \sin\left(\theta \right)\sin\left(\phi \right) & \cos\left(\phi \right)\\ -\sin\left(\theta \right) & \cos\left(\theta \right) & 0\\ -\cos\left(\theta \right)\cos\left(\phi \right) & -\sin\left(\theta \right)\cos\left(\phi \right) & \sin\left(\phi \right) \end{array}\right).$$

**Figure 7:**
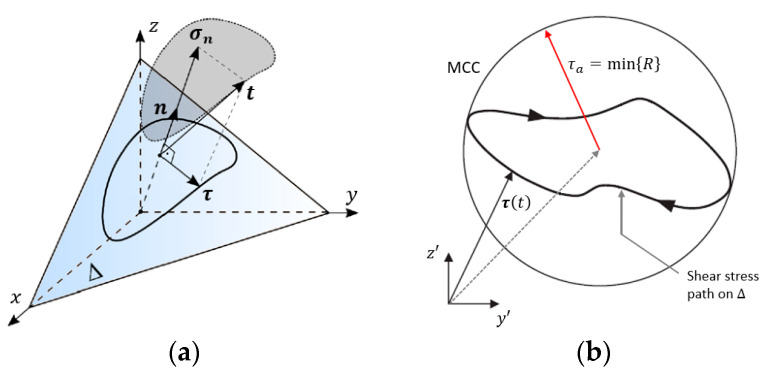
(**a**) Stress vector t decomposed into normal and shear stress components $\sigma_{n}$ and τ, with the latter establishing a shear stress path on Δ; (**b**) MCC procedure to estimate the shear stress amplitude $\tau_{a}$ acting on a material plane Δ.

The determination of $\sigma_{n,max}$ acting on the material plane Δ is usually simple, as it just corresponds to the maximum value attained by $\sigma_{n}(t)$ within a loading cycle. On the other hand, determining the shear stress amplitude $\tau_{a}$ acting on the material plane Δ can be challenging, as the shear stress vector $\tau \left(t\right)$ may vary both in magnitude and in direction throughout a loading cycle [7], as shown in Figure 7a. A popular approach to determine the shear stress amplitude $\tau_{a}$ is known as the minimum circumscribed circle (MCC) [12,13], which consists of identifying the trajectory established by the shear stress vector on the material plane Δ and circumscribing it with a circle, as presented in Figure 7b. The shear stress amplitude acting on the material plane in question is taken as the radius of the MCC.

#### 2.1.1. Application Procedure

The present work selected four critical-plane stress-based multiaxial high-cycle fatigue criteria, namely Findley (F), Matake (M), McDiarmid (McD) and Susmel and Lazzarin (S&L). The expressions corresponding to the four models respectively are given in [4,5,6,7]

(6) $$\tau_{a}^{*}+\kappa \;\sigma_{n,max}^{*}\;\leq \lambda$$

(7) $$\tau_{a}^{*}+\mu \;\sigma_{n,max}^{*}\leq \tau_{-1}$$

(8) $$\tau_{a}^{*}+\frac{\tau_{-1}}{2\;\sigma_{u}}\;\sigma_{n,max}^{*}\leq \tau_{-1}$$

(9) $$\tau_{a}^{*}+\left[\tau_{-1}-\frac{\sigma_{-1}}{2}\right]\frac{\sigma_{n,max}^{*}}{\tau_{a}^{*}}\;\leq \tau_{-1},$$

where $\tau_{a}^{*}$ and $\sigma_{n,max}^{*}$, respectively, correspond to the shear stress amplitude and the maximum value attained by the normal stress acting on the critical planes. The parameters $\sigma_{-1}$ and $\tau_{-1}$ are the fatigue resistance limits in fully reversed push–pull and pure torsion tests. The constant $\sigma_{u}$ corresponds to the ultimate tensile strength, while the remaining quantities κ, λ and μ are constants that can be directly evaluated from $\sigma_{-1}$ and $\tau_{-1}$, as given in [4,5]

(10) $$\kappa =\frac{2-\left(\frac{\sigma_{-1}}{\tau_{-1}}\right)}{2\sqrt{\frac{\sigma_{-1}}{\tau_{-1}}-1}}$$

(11) $$\lambda =\;\sqrt{\frac{\sigma_{-1}^{2}}{4\left(\frac{\sigma_{-1}}{\tau_{-1}}-1\right)}}$$

(12) $$\mu =\;2\left(\frac{\tau_{-1}}{\sigma_{-1}}\right)-1.$$

As for critical plane definition, Findley takes into account the combined effect of $\tau_{a}$ and $\sigma_{n,max}$, defining as critical the one or more planes that maximise the linear combination of $\tau_{a}+\kappa \;\sigma_{n,max}$ [4]. Matake, McDiarmid and Susmel and Lazzarin consider that the shear stress amplitude $\tau_{a}$ plays the most important role in crack nucleation. As such, the one or more planes that experience the highest value of shear stress amplitude are classified as candidate planes. The normal stress, in turn, plays an additional role, as tensile stresses tend to separate crack surfaces, thus shortening fatigue life. Therefore, the critical planes correspond to those where the maximum value of the normal stress $\sigma_{n,max}$ is experienced within a set of candidate planes where the shear stress amplitude $\tau_{a}$ had already been found to be maximum [5,6,7].

Expressions (2)–(4) were applied to the loading histories resulting from the FEM analysis with the purpose of obtaining $\sigma_{n}\left(t\right)$, $\tau_{x^{’}y^{’}}\left(t\right)$ and $\tau_{x^{’}z^{’}}\left(t\right)$ for a number of material planes. A computational procedure was implemented considering increments of Δθ corresponding to 1° and of Δϕ corresponding to 5°, with θ ranging from 0° to 179° and ϕ ranging from 0° to 175°. The quantities $\sigma_{n,max}$ and $\tau_{a}$ were computed for the material planes in question, with $\tau_{a}$ being determined via MCC. As result, the critical values of $\tau_{a}^{*}$ and $\sigma_{n,max}^{*}$ relative to each model were determined, allowing the application of the involved criteria. Accordingly, the procedure was carried out for each one of the twenty critical points of the crankshaft.

#### 2.1.2. Estimating $\sigma_{-1}$ and $\tau_{-1}$

Ferrous materials often present a certain threshold known as the fatigue resistance limit. If the applied stress amplitude is inferior to this reference threshold, failure is not expected to occur, thus leading to a theoretically infinite fatigue life. Nevertheless, determining the fatigue resistance limits can be challenging and time-consuming so, instead, it is common practice to adopt the concept of endurance limit, which corresponds to the stress amplitude extrapolated in the high-cycle fatigue regime, associated with a given number of cycles to failure $N_{ref}$ [14].

In this particular operation, the motor engine operates at 720 rpm, where, as previously mentioned, an entire loading cycle corresponds to two revolutions of the crankshaft. Consequently, the crankshaft experienced 360 cycles within one minute of operation, which corresponds to 6 Hz. Considering that each motor engine is designed to operate for 6 months a year for 25 years, the number of cycles to failure taken as a reference for the endurance limits can be estimated as

(13) $$N_{ref}=6\frac{cycles}{s}\times 3600\frac{s}{h}\times 24\frac{h}{day}\times \left(\frac{365}{2}\right)\frac{day}{year}\times 25\;years\;N_{ref}\approx 2.4\times 10^{9}\;cycles.$$

Considering that the corresponding Wöhler curves associated with the DIN 34CrNiMo6 steel in push–pull and pure torsion are, respectively, given by [15,16]

(14) $$\sigma_{a}=1470.4\;N^{-0.062}$$

(15) $$\tau_{a}=834.1\;N^{-0.047},$$

the endurance limits can be accordingly determined.

(16) $$\sigma_{-1}=1470.4\;N_{ref}^{-0.062}\approx 1470.4\;{\left(2.4\times 10^{9}\right)}^{-0.062}\approx 385\;MPa$$

(17) $$\tau_{-1}=834.1\;N_{ref}^{-0.047}\approx 834.1\;{\left(2.4\times 10^{9}\right)}^{-0.047}\approx 302\;MPa$$

Finally, the fatigue behaviour of the DIN 34CrNiMo6 is assessed using the error index, as defined in [8]

(18) $$I=\frac{LHS-RHS}{RHS},$$

which compares the left-hand side (LHS) of expressions (6)–(9) with their corresponding right-hand sides (RHSs). Considering that the LHS is associated with the driving force to failure, while the RHS is associated with fatigue resistance limits, positive I values indicate that the driving force to failure exceeds fatigue resistance limits and therefore specimen cracking is to be expected. On the other hand, negative I values indicate that the fatigue resistance limits are greater than the driving force to failure, thus implying that fatigue failures should not occur.

### 2.2. Determination of $\Delta K_{th}$ and Experimental Procedure

As reported in the literature [17,18,19,20,21,22], the crack growth rate is controlled by the applied stress intensity factor range ΔK. However, the application of ΔK values inferior to a certain threshold results in the formation of non-propagating cracks arrested by microstructural barriers [14,17].

On that account, the stress intensity threshold range $\Delta K_{th}$ arises as a quantity of interest, as it represents a measure of the material’s resistance to crack growth. In addition to the DIN 34CrNiMo6 steel, three commercially available steels (DIN 42CrMo4, SAE 4140 and SAE 4340), comparable to the DIN 34CrNiMo6 in structural applications, were also taken into consideration. The crack growth thresholds ($\Delta K_{th}$) of all the involved materials were determined and compared, aiming to correlate the $\Delta K_{th}$ values with the metallurgical impurity contents of their respective steels. The goal was to verify whether similar commercial steels presented a higher or lower number of inclusions and, consequently, assess the influence of the impurity content on the respective crack propagation rates.

**Figure 8:**
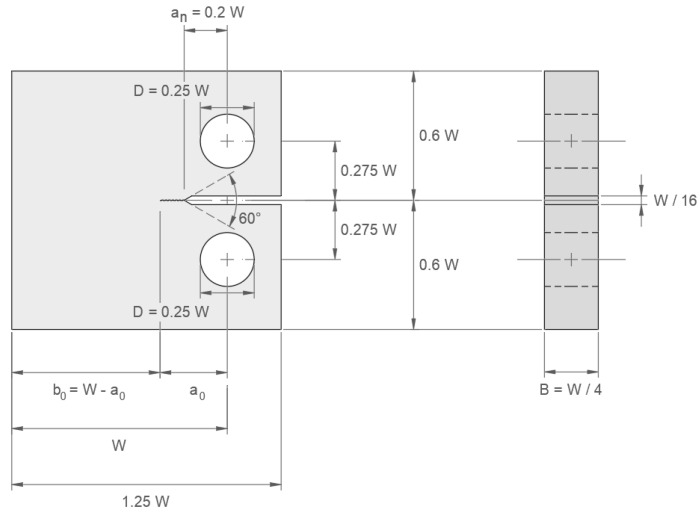
CT specimen used in the experimental research.

As illustrated in Figure 8, the compact tension (CT) specimens used in the experimental work were manufactured according to an international standard [23], and their dimensions are presented in Table 4. However, it is important to note that the $\Delta K_{th}$ determined under tensile loading is higher than that determined under multiaxial loading, resulting in higher fatigue threshold values. This indicates that the materials exhibit greater resistance to crack propagation when subjected to uniaxial tension compared to mixed-mode loading [24,25].

#### 2.2.1. Precracking and Specimen Preparation

Specimen precracking is to be carried out using sinusoidal fatigue loadings [23], which are reported as being generally most effective when applying a load ratio R, defined as $P_{min}/P_{max}$, of 0.1 [26,27]. Considering the specimen dimensions presented in Table 4, fatigue loading is applied until precrack extension equals or exceeds 1.3 mm [23], which usually requires the application of $10^{4}$ to $10^{6}$ loading cycles [26].

The applied load range ΔP and the stress intensity factor range ΔK, defined as $P_{max}-P_{min}$ and $K_{max}-K_{min}$, can be related, as given in [23,27]

(19) $$\Delta K=\frac{\Delta P}{{\left(B^{2}W\right)}^{1/2}}\;f\left(\frac{a}{W}\right),$$

where

(20) $$f\left(\frac{a}{W}\right)=\frac{\left(2+\frac{a}{W}\right)\;}{{\left(1-\frac{a}{W}\right)}^{3/2}}\left[0.886+4.64\left(\frac{a}{W}\right)-13.32{\left(\frac{a}{W}\right)}^{2}+14.72{\left(\frac{a}{W}\right)}^{3}-5.6{\left(\frac{a}{W}\right)}^{4}\right].$$

As presented in Figure 8, the parameters W and B correspond to the specimen’s width and thickness, where the latter is set to be W/4. Prior to precracking, a corresponds to the notch length $a_{n}$, measured along the notch bisector from the notch apex to the centreline of the holes, with $a_{n}$ corresponding to 0.2 W. Once a precrack has developed, a must be accordingly increased by the existing precrack length, and the total length is herein denoted as $a_{0}$.

By considering the maximum values of $P_{max}$ and $K_{max}$ and by including the influence of a developed precrack, expression (19) takes the form

(21) $$K_{max}=\frac{P_{max}}{{\left(B^{2}W\right)}^{1/2}}\;f\left(\frac{a_{0}}{W}\right).$$

For a $K_{max}$ of $25\;MPa\;m^{1/2}$ [28] and a final precrack length of 1.5 mm, precracking loads $P_{max}$ and $P_{min}$ were found to be approximately 15 kN and 1.5 kN for the larger specimens and 11 kN and 1.1 kN for the smaller specimens, resulting in a ΔK of $22.5\;MPa\;m^{1/2}$.

Crack size measurements must be carried out using a technique capable of resolving crack extensions of 0.10 mm. As per the standard [23], the present study employed visual measurement with the use of a 50× travelling optical microscope. A length scale was attached to the specimens, and the crack length was periodically observed and registered.

Specimen preparation involved polishing the test surface to a mirror-like finish, initially with 100–1200 sandpaper grit and eventually proceeding to a metallographic polishing machine using synthetic cloth pads and diamond polishing pastes with abrasive particles of 6, 3 and 1μm.

#### 2.2.2. Fatigue Crack Growth Threshold $\Delta K_{th}$ Determination Procedure

**Figure 9:**
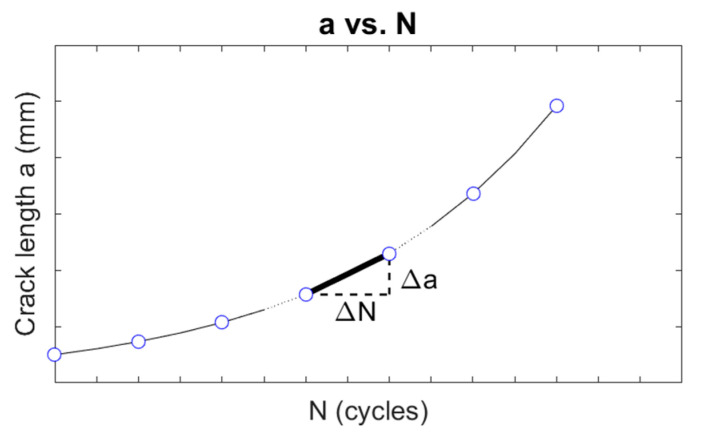
Crack growth rates obtained from the a versus N curve.

The fatigue crack growth threshold can be obtained from experiments where the crack growth rate da/dN is plotted against the stress intensity range ΔK. Based on this, the crack growth rate can be determined using the secant method [23], which consists of calculating the slope of the tangent line between two adjacent data points on the a versus N curve, as shown in Figure 9.

Accordingly, for the i-th measurement, da/dN can be obtained, as given by [23]

(22) $${\left.\frac{da}{dN}\;\right|}_{i}=\frac{a_{i+1}-a_{i}}{N_{i+1}-N_{i}}.$$

As for the stress intensity factor range, $\Delta K_{i}$ can be obtained by employing expression (19), which takes the form [23,26]

(23) $$\Delta K_{i}=\frac{\Delta P_{i}}{{\left(B^{2}W\right)}^{1/2}}\;f\left(\frac{a_{i}}{W}\right).$$

The determination of $\Delta K_{th}$ is carried out by gradually reducing the stress intensity range ΔK up to a point where crack propagation eventually stops. The first value of $P_{max}$ can be determined using expression (23) for a crack length of $a_{0}$. Two reference values of ΔK were taken into consideration, following the recommendations provided by two different standards [27,28]. The latter, as discussed in the precracking stage, proposed a $\Delta K^{\left(1\right)}$ of $22.5\;MPa\;m^{1/2}$. The former, in turn, proposes that the relation between the maximum stress intensity factor and the elastic modulus $K_{max}/E$ must be kept under $0.0003\;m^{1/2}$. By adopting 60% of such a value (with the purpose of distancing from limiting values), $\Delta K^{\left(2\right)}$ turns out to be approximately $33.5\;MPa\;m^{1/2}$. While loads leading to ΔK values between $\Delta K^{\left(1\right)}$ and $\Delta K^{\left(2\right)}$ were considered to be acceptable, the present work aimed at $28\;MPa\;m^{1/2}$, as it constitutes an average value between $\Delta K^{\left(1\right)}$ and $\Delta K^{\left(2\right)}$. Accordingly, the initial loads, $P_{max}$ and $P_{min}$, applied in the experiments are presented in Table 5.

Specimens were then subjected to fatigue loadings, where the crack extension was measured and registered after $5\times 10^{3}$ to $10^{4}$ loading cycles. The procedure is continuously repeated, each time decreasing the loads by 10% until the crack did not present any further propagation after $10^{5}$ cycles.

At this point, loads should be slightly increased (3 to 5%), with the purpose of verifying if the last reduction step was not excessive. If crack growth is resumed, this indicates that the previous ΔK corresponded to the $\Delta K_{th}$. Otherwise, if crack extension holds when the specimen is subjected to slightly increased loads, ΔK may be further increased until the crack resumes its propagation. In this case, the last ΔK before crack propagation resumes can be taken as $\Delta K_{th}$.

## 3. Results and Discussion

### 3.1. Multiaxial Fatigue Analysis

The selected multiaxial stress-based high-cycle fatigue criteria were applied to the time-varying states of stress developed in twenty critical locations (critical points) of the crankshaft. The fatigue behaviour is thus assessed via error indices, as defined in expression (18).

**Figure 10:**
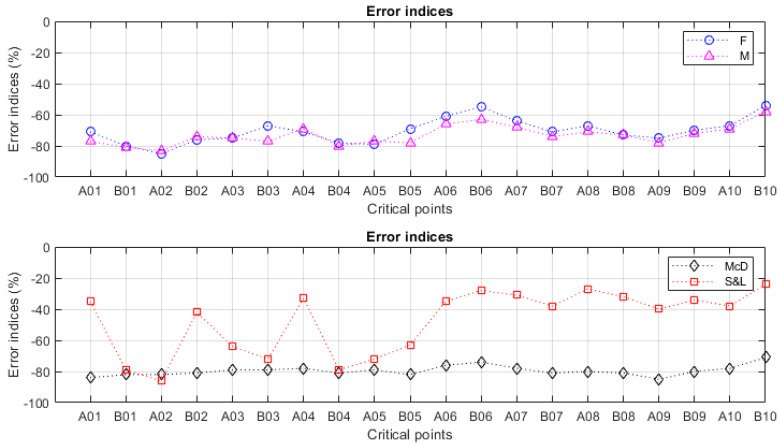
Error indices resulting from the multiaxial fatigue analysis.

The results of the multiaxial fatigue analysis are presented in Figure 10 and in Table 6, revealing that all the error indices were found to be negative. This indicates that the stresses developed in critical points of the crankshaft did not exceed the material’s fatigue resistance and, therefore, should not drive the component to failure.

Findley provided error indices ranging from −85% to −54%, while Matake outputted indices between −83% and −58%. As one may see from Figure 10, the error index distributions associated with these two models were found to be similar, and the figures involved indicate that that the crankshaft’s operation was considerably far from failure. In terms of the average and standard deviation, Findley reported an average of −70.4%, with a standard deviation of 7.7%, while Matake’s average was −73.2%, with a corresponding standard deviation of 6.1%.

McDiarmid presented a flat distribution, where the indices were found to be even more negative (thus indicating safety) within a relatively narrow range from −85% to −71%. The corresponding average and standard deviation were, respectively, revealed to be −79.6% and 3.1%.

In agreement with Findley, Matake and McDiarmid, the error indices obtained from Susmel and Lazzarin (S&L) were also found to be negative. Nevertheless, in comparison to the other models, the resulting values were found to be higher, especially for the critical points B06, A07, A08 and B10, where the error indices, respectively, corresponded to −28%, −31%, −27% and −24%. At this point, it is important to verify the significance of these values, assessing how far the crankshaft is from failure when subjected to such loading conditions. In practice, the following discussion consists of verifying the influence of an overestimation of the endurance limits or an underestimation of the applied stresses over S&L’s predictions.

**Figure 11:**
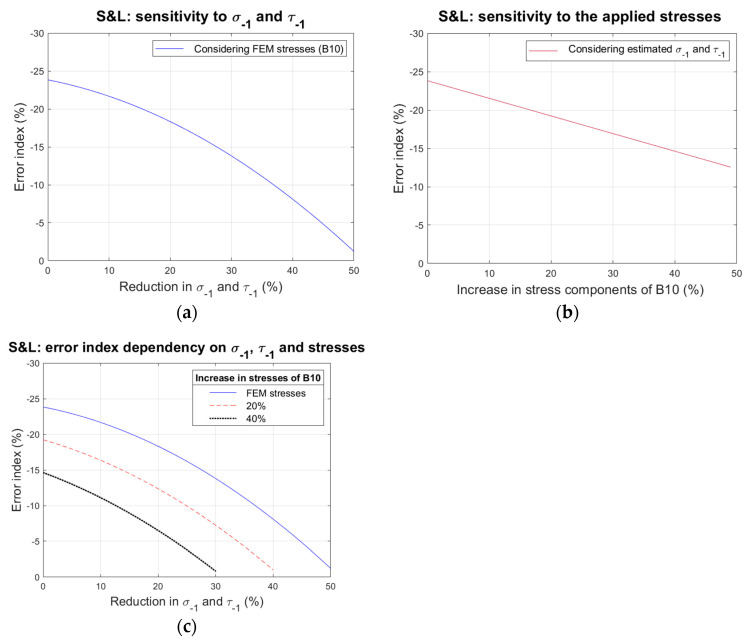
Sensitivity of the error index with respect to (**a**) reductions in $\sigma_{-1}$ and $\tau_{-1}$, (**b**) increases in applied stresses; and (**c**) both events occurring simultaneously.

Overestimating fatigue limits would result in non-conservative predictions, as it would lead to an interpretation where stresses could be raised while maintaining safe operating conditions. By taking into consideration the loading history associated with B10, the sensitivity of the S&L criterion to $\sigma_{-1}$ and $\tau_{-1}$ was investigated, revealing that the magnitude of the error index decreases with reductions in $\sigma_{-1}$ and $\tau_{-1}$ according to a quadratic relationship, as presented in Figure 11a. Interestingly enough, the S&L criterion maintains its indication of safety even when significant reductions in $\sigma_{-1}$ and $\tau_{-1}$ were considered. For instance, simultaneously halving $\sigma_{-1}$ and $\tau_{-1}$ leads to values of $\sigma_{-1}^{’}$ and $\tau_{-1}^{’}$ corresponding to approximately 193 MPa and 151 MPa, resulting in an error index of −0.83%, which, in practice, is very close to the limiting state of non-fracture but still on the safe side.

In addition, the effect of underestimating the applied stresses was also investigated. By applying amplified versions of the loading history associated with B10 to the S&L criterion, a linear dependency of the error index with respect to the amplification factor was revealed, as presented in Figure 11b. Likewise, the analysis revealed that the S&L criterion once again maintains its indication of safety, even for relatively high amplification factors. In this case, doubling the stresses associated with B10 results in the same error index value of −0.83%.

Finally, it is important to mention that both events (overestimating the fatigue limits and underestimating the stresses) could occur simultaneously. Figure 11c illustrates the influence of both events taking place simultaneously over S&L’s predictions. Accordingly, the same limiting state of non-fracture (error index of −0.83%) can be obtained by reducing $\sigma_{-1}$ and $\tau_{-1}$ by 40% while amplifying the stresses by 20% or by reducing $\sigma_{-1}$ and $\tau_{-1}$ by 30% while amplifying the stresses by 40%.

As a result, it is concluded that even though the S&L results were revealed to be more conservative, the stresses developed during the operation in the critical points of the crankshaft were not sufficient to drive the component to failure.

### 3.2. Results Involving the Fatigue Crack Growth Threshold $\Delta K_{th}$

Crack growth experiments were carried out with the purpose of determining the $\Delta K_{th}$ of the DIN 34CrNiMo6 steel, following the experimental procedure described in Section 2.2 of the present work. The test data, registered throughout the experiment, is summarised and presented in Table 7.

As one may observe, the crack ceased to propagate in the penultimate step, when $\Delta K_{i}$ was reduced from 7.12 to $6.4\;MPa\;m^{1/2}$. Aiming to verify if that a 10% reduction was not excessive, an increment of 3% in ΔK was applied (raising ΔK to $6.6\;MPa\;m^{1/2}$), and crack length remained unaltered. Nevertheless, crack growth resumed when further increases in ΔK were applied, implying that $6.6\;MPa\;m^{1/2}$ is a reasonably precise measure of the DIN 34CrNiMo6 steel stress intensity threshold range $\Delta K_{th}$.

**Figure 12:**
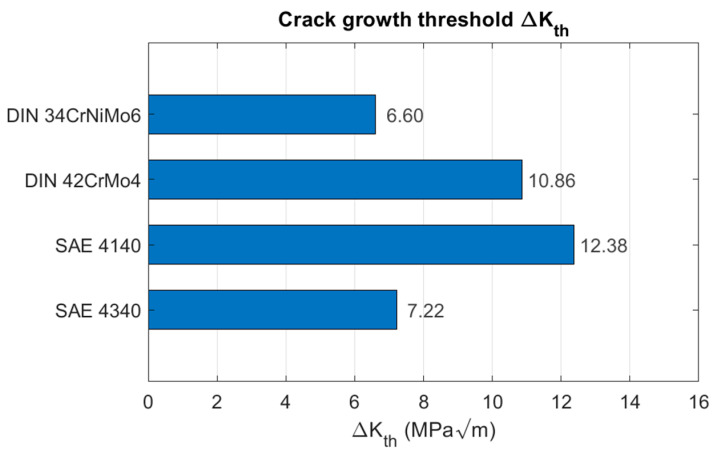
Comparison between the crack growth thresholds of the involved steels.

Following the same experimental procedure, the values of $\Delta K_{th}$ measured for the other involved steels were also determined. The results are presented in Figure 12 in a comparative manner, including the result obtained for the DIN 34CrNiMo6 steel.

As one may observe, the $\Delta K_{th}$ value measured for the DIN 34CrNiMo6 steel (the focus of the present work) was the lowest value among all the considered steels. The measured value of $6.60\;MPa\;m^{1/2}$ is only 4% higher than the value recommended by Det Norske Veritas $\left(6.32\;MPa\;m^{1/2}\right)$ for guarding against fatigue using structural steels [22]. Considering that this quantity represents a measure of the material’s resistance to crack propagation, it can be concluded that newly developed cracks will tend to propagate sooner and with higher growth rates in DIN 34CrNiMo6, therefore this material is more susceptible to shorter fatigue lives in comparison with the other steels considered in this work.

Although a fatigue analysis of the material was conducted in terms of service stresses and design criteria, this analysis did not clarify the cause of the failure. On the other hand, microstructural analysis of the material indicated a large number of inclusions, which in turn impacted the crack propagation rate.

### 3.3. Complementary Analysis Regarding the Impurity Content of the Materials

It is well-established in the literature that the fatigue crack growth threshold is sensitive to metallurgical factors [14,22] and is therefore influenced by the presence of metallurgical defects. The materials were thus analysed for their impurity content using scanning electron microscopy (SEM).

**Figure 13:**
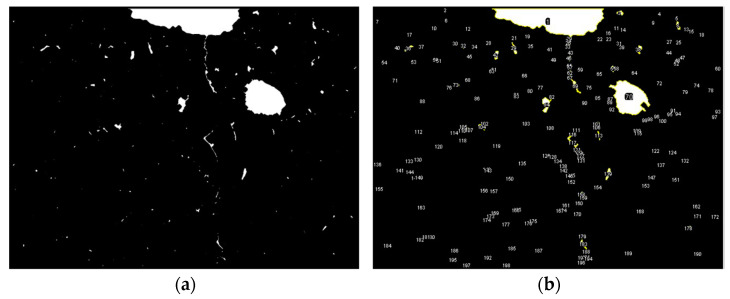
(**a**) Segmented image corresponding to an area of 200 × 200 μm^2^ relative to the DIN 34CrNiMO6 steel; (**b**) particle identification and numbering.

SEM images of the DIN 34CrNiMo6 steel were obtained, as exemplified in Figure 13a, and subsequently segmented. With the use of a commercial digital image processing software, the particles were identified and numbered, as shown in Figure 13b. This procedure was repeated five times at random locations within the material sample, revealing an impurity content of 550 particles/mm^2^ for this particular material.

**Figure 14:**
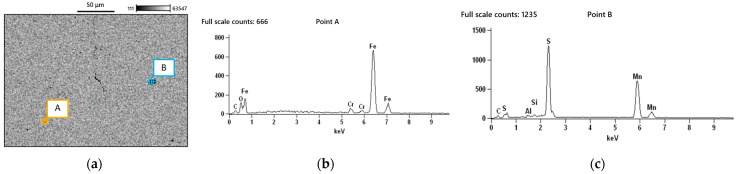
(**a**) SEM image of the specimen, where impurities A and B were examined; (**b**) particle A, revealing the presence of oxides; (**c**) particle B, revealing the presence of sulphides.

Accordingly, the particles were analysed using energy dispersive X-ray spectroscopy (EDS) in order to determine their chemical compositions. Figure 14a shows an SEM image of the specimen, where impurities A and B were examined. As shown in Figure 14b for particle A, EDS revealed the presence of the expected alloying elements, along with a certain amount of oxygen, indicating the presence of oxides. In the case of particle B, EDS identified the presence of sulphides.

**Figure 15:**
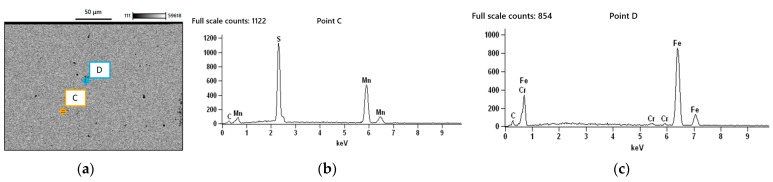
(**a**) SEM image of another region within the specimen, where impurities C and D were examined; (**b**) particle C, revealing the presence of sulphides; (**c**) particle D, revealing voids.

In addition, Figure 15a presents an SEM image of another region within the specimen, which similarly contains a couple of impurities (C and D). As shown in Figure 15b, EDS revealed once again presence of sulphides in particle C, whereas Figure 15c indicates the presence of voids associated with particle D.

Finally, this procedure was repeated for all the involved materials, revealing that DIN 34CrNiMo6—which presented the lowest fatigue crack growth threshold $\Delta K_{th}$—was found to have a significantly higher impurity content in comparison to the other steels. The results are summarised in Table 8.

## 4. Conclusions

The present work represents an investigation into whether the stresses developed in critical locations of a crankshaft forged from DIN 34CrNiMo6 steel operating in a thermoelectric power plant environment were in accordance with the material’s fatigue limits. An additional investigation was carried out to determine whether the material presented an adequate resistance to crack propagation. In this sense, a set of conclusions can be drawn:

- The loading histories were applied to four critical plane-based high-cycle fatigue models, namely Findley (F), Matake (M), McDiarmid (McD) and Susmel and Lazzarin (S&L). The fatigue behaviour was assessed via error indices. In every single case, the models unanimously indicated that the stresses were not sufficient to drive the component to failure, thus indicating safety.
- Nevertheless, the error indices relative to the S&L criterion, while still indicating safety, were revealed to be higher in comparison to those delivered by the other models. Taking into consideration the loading conditions associated with the highest indices, an additional investigation was carried out with the purpose of assessing, in practice, how far the component was from failure. The analysis revealed that the S&L criterion would maintain its prediction of safety even if one were to double the magnitude of the involved stresses or to reduce the fatigue limits considered in this work by 50%. As such, one may conclude that these higher indices are associated with a certain conservativeness in the S&L criterion, thus supporting the perception that the stresses developed in the operation were adequate for this particular material.
- Experimental research was carried out to determine the crack growth thresholds $\Delta K_{th}$ of DIN 34CrNiMo6, as well as for three other commercially available steels, namely DIN 42CrMo4, SAE 4140 and SAE 4340. The results revealed that the steel from which the crankshaft was forged (DIN 34CrNiMo6) presented the lowest value of $\Delta K_{th}$ among all the considered steels, thus indicating that it has the least resistance to crack propagation and is therefore susceptible to a shorter fatigue life in comparison to the other considered steels.
- The research showed that the crankshaft failure was not caused by operating stresses or design errors. The evidence points to the importance of the of the impurity content in the material, which negatively impacts the nucleation and propagation of fatigue cracks.

## Figures and Tables

**Table 1:** Chemical compositions of the DIN 34CrNiMo6, DIN 42CrMo4, SAE 4140 and SAE 4340 steels.

Steel	Fe (%)	C (%)	Mn (%)	Si (%)	Cu (%)	Cr (%)	V (%)	Mo (%)	Ni (%)	Others (%)
DIN 34CrNiMo6	95.1	0.36	0.52	0.24	--	1.50	--	0.24	1.72	0.32
DIN 42CrMo4	96.9	0.38	0.85	0.27	0.18	0.97	0.01	0.2	--	0.24
SAE 4140	97.1	0.42	0.86	0.26	0.01	1.06	0.0047	0.17	0.043	0.0723
SAE 4340	96.2	0.42	0.64	0.23	0.16	0.75	0.024	0.21	1.26	0.106

**Table 2:** Mechanical properties of the DIN 34CrNiMo6, DIN 42CrMo4, SAE 4140 and SAE 4340 steels.

Steel	$\sigma_{y}\left(MPa\right)$	$\sigma_{u}\left(MPa\right)$	Hardness
DIN 34CrNiMo6	728	897	302 HV
DIN 42CrMo4	689	861	320 HV
SAE 4140	587	802	262 HB
SAE 4340	675	845	277 HB

**Table 3:** Representation of the estimated stress fields resulting from the FEM analysis relative to critical point A06, where each time unit corresponding to approximately $2.315\times 10^{-4}s$.

Angular Position (°) or Time Units	$\sigma_{xx}$ $\left(MPa\right)$	$\sigma_{yy}$ $\left(MPa\right)$	$\sigma_{zz}$ $\left(MPa\right)$	$\tau_{xy}$ $\left(MPa\right)$	$\tau_{yz}$ $\left(MPa\right)$	$\tau_{xz}$ $\left(MPa\right)$
0	−8.02	−6.52	−42.69	−4.58	48.56	28.07
1	−8.15	−7.00	−43.87	−4.64	47.09	27.60
2	−8.29	−7.48	−45.08	−4.71	45.68	27.15
3	−8.44	−7.97	−46.32	−4.78	44.35	26.75
4	−8.59	−8.45	−47.60	−4.85	43.09	26.39
⋮	⋮	⋮	⋮	⋮	⋮	⋮
715	−7.45	−4.16	−37.22	−4.31	56.62	30.86
716	−7.55	−4.62	−38.26	−4.36	54.95	30.27
717	−7.66	−5.09	−39.32	−4.41	53.30	29.69
718	−7.77	−5.56	−40.41	−4.47	51.66	29.12
719	−7.77	−5.56	−40.41	−4.47	51.66	29.12

**Table 4:** CT specimen dimensions (in millimetres).

Steel	$W\left(mm\right)$	$B\left(mm\right)$	$a_{n}\left(mm\right)$	$b_{0}\left(mm\right)$
DIN 34CrNiMo6	50	12.5	10	40
DIN 42CrMo4	50	12.5	10	40
SAE 4140	40	10	8	32
SAE 4340	40	10	8	32

**Table 5:** Initial loads for the da/dN versus ΔK experiments.

Steel	$P_{max}\left(kN\right)$	$P_{min}\left(kN\right)$	R
DIN 34CrNiMo6	20.12	1.998	0.10
DIN 42CrMo4	19.20	1.890	0.10
SAE 4140	13.65	1.353	0.10
SAE 4340	13.53	1.480	0.11

**Table 6:** Resulting error indices (multiaxial fatigue).

Loading Conditions	$F\left(\%\right)$	$M\left(\%\right)$	$McD\left(\%\right)$	$S\&L\left(\%\right)$
A01	−71	−77	−84	−35
B01	−80	−81	−82	−79
A02	−85	−83	−82	−86
B02	−76	−74	−81	−42
A03	−75	−75	−79	−64
B03	−67	−77	−79	−72
A04	−71	−69	−78	−33
B04	−78	−80	−81	−79
A05	−79	−77	−79	−72
B05	−69	−78	−82	−63
A06	−61	−66	−76	−35
B06	−55	−63	−74	−28
A07	−64	−68	−78	−31
B07	−71	−74	−81	−38
A08	−67	−71	−80	−27
B08	−73	−73	−81	−32
A09	−75	−78	−85	−40
B09	−70	−72	−80	−34
A10	−67	−69	−78	−38
B10	−54	−58	−71	−24

**Table 7:** Test data registered throughout the experiment.

$P_{max}$ $\left(N\right)$	$P_{min}$ $\left(N\right)$	$a_{i}$ $\left(mm\right)$	Δa $\left(mm\right)$	ΔN $\left(cycles\right)$	$\Delta K_{i}$ $\left(MPa\sqrt{m}\right)$	da/dN $\left(mm/cycle\right)$
20,120	1998	1.6	1.0	9000	31.07	1.11 × 10^−4^
18,450	1890	2.6	0.7	6250	29.34	1.12 × 10^−4^
16,790	1653	3.3	0.65	7220	27.84	9.00 × 10^−5^
15,200	1517	3.95	0.6	5076	26.05	1.18 × 10^−4^
13,290	1355	4.55	0.55	5810	23.45	9.47 × 10^−5^
11,790	1147	5.1	0.45	6580	21.51	6.84 × 10^−5^
10,550	1047	5.55	0.45	5650	19.66	7.96 × 10^−5^
9441	945	6	0.45	6590	17.99	6.83 × 10^−5^
8345	830	6.45	0.35	9300	16.28	3.76 × 10^−5^
7500	743	6.8	0.3	6500	14.91	4.62 × 10^−5^
6750	685	7.1	0.35	7180	13.59	4.87 × 10^−5^
6095	609	7.45	0.3	6010	12.51	4.99 × 10^−5^
5475	558	7.75	0.3	5440	11.39	5.51 × 10^−5^
4937	496	8.05	0.25	5660	10.45	4.42 × 10^−5^
4499	444	8.3	0.25	6483	9.66	3.86 × 10^−5^
3954	393	8.55	0.15	6850	8.60	2.19 × 10^−5^
3614	363	8.7	0.05	7085	7.91	7.06 × 10^−6^
3245	325	8.75	0.05	7310	7.12	6.84 × 10^−6^
2920	302	8.8	0	100,010	6.40	0
3002	301	8.8	0	100,000	6.60	0

**Table 8:** Correlation between the experimentally measured fatigue crack growth thresholds $\Delta K_{th}$ and the corresponding population of metallurgical defects.

Steel	$\Delta K_{th}\left(MPa\sqrt{m}\right)$	Impurity Content (particles/mm^2^)
DIN 34CrNiMo6	6.60	550
DIN 42CrMo4	10.86	95
SAE 4140	12.38	125
SAE 4340	7.22	110

## Data Availability

The original contributions presented in this study are included in the article. Further inquiries can be directed to the corresponding author.
